# Community-Acquired *Chlamydia psittaci* Severe Pneumonia: A Case Report

**DOI:** 10.1155/crdi/6627159

**Published:** 2025-09-04

**Authors:** Quentin Guillemot, Thomas Clemens, Valentine Inthasot, Bhavna Mahadeb, Evelyne Maillart, Philippe Clevenbergh

**Affiliations:** ^1^Internal Medicine Department, University Hospital Brugmann, Free University Brussels, Brussels, Belgium; ^2^Pneumology Department, University Hospital Brugmann, Free University Brussels, Brussels, Belgium; ^3^Department of Microbiology, University Hospital Brugmann, Free University Brussels, Brussels, Belgium; ^4^Clinic of Infectious Diseases, University Hospital Brugmann, Free University Brussels, Brussels, Belgium

**Keywords:** atypical pneumonia, *Chlamydia psittaci*, community-acquired pneumonia, multiplex PCR respiratory panel, zoonosis

## Abstract

*Chlamydia psittaci*, the causative agent of psittacosis, is an intracellular bacterium typically transmitted from birds to humans, leading to atypical pneumonia. We present a case of a 60-year-old man with no reported bird exposure but a history of working as a chief cook, potentially exposed to poultry. He presented with high fever, diffuse soreness, and left-sided pulmonary consolidation. Initial treatment with β-lactams was ineffective, but a multiplex PCR on bronchoalveolar lavage identified *C. psittaci* DNA. Therapy was switched to moxifloxacin, resulting in rapid clinical improvement. *C. psittaci* causes approximately 1% of community-acquired pneumonias, often underdiagnosed due to nonspecific symptoms and the need for advanced diagnostic tools like nucleic acid amplification tests (NAATs) or metagenomic next-generation sequencing (mNGS). The bacterium is endemic in birds and poultry, with human infections linked to occupational exposure or contact with infected animals. Diagnosis relies on NAAT and mNGS, as serology and culture are less practical. Treatment with tetracyclines, quinolones, or macrolides is effective, reducing mortality from 10%–20% to < 1%. Preventive measures, including protective equipment for high-risk individuals and treatment of infected birds, are crucial. Mandatory reporting of cases could improve understanding of the disease burden. This case highlights the importance of considering psittacosis in atypical pneumonia, even without direct bird exposure, and the role of NAAT or mNGS in accurate diagnosis.

## 1. Introduction

Pneumonia is the leading cause of infection-associated death worldwide [1]. The place of acquisition of the pneumonia (healthcare-associated, nosocomial, or community-acquired) and the underlying conditions direct empiric antibiotherapy [2]. Within the group of community-acquired pneumonia (CAP), we can diagnose “classic” pneumonia from “atypical” pneumonia [3]. “Classic” pneumonias are predominantly caused by *S. pneumoniae*, *H. influenza*, *M. catarrhalis*, or *S. aureus*, often sensitive to b-lactams. “Atypical” pneumonias, by definition, do not respond to b-lactams. There are no bacteria at Gram stain sputum examination because the causative agents are intracellular bacteria or viruses. Bacterial atypical pneumonia can further be classified into non-zoonotic and zoonotic bacterial pneumonia. The non-zoonotic “atypical” pneumonias are due to *L. pneumophila*, *M. pneumoniae*, and *C. pneumoniae*. Zoonotic atypical pneumonias are due to *C. burnetii*, *C. psittaci*, or *F. tularensis* [3]. We report such a case of atypical pneumonia due to *C. psittaci* diagnosed by multiplex PCR respiratory panel.

## 2. Case

**Figure 1:**
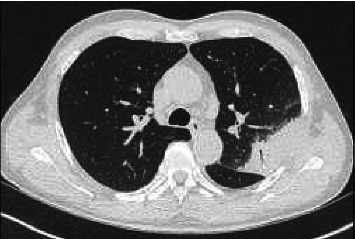
Chest CT scan showing subpleural lobar condensation in the left upper lobe with air bronchogram.

**Figure 2:**
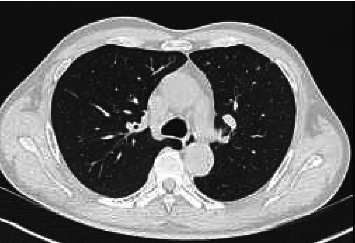
Two months later, chest CT scan showing complete resolution of infiltrate from posterior pneumonia of the upper left lobe.

Our patient is a 60-year-old man, born in China. He was complaining of diffuse soreness and high fever. He came back from a two-month trip in China, 2 months earlier. He reported no contact with another sick person. He had no other complaints. His medical history consists of type 2 diabetes and high blood pressure treated with metformin and perindopril/amlodipine. On clinical examination, we observed the following: temperature at 40.1°C, respiratory rate 15/min, heart rate 101/min, blood pressure 146/85 mmHg, O2 saturation 97% at room air, and inspiratory crackles in the left lung. The initial blood test showed white blood cells of 10,770 cells/μL, neutrophils of 83%, C-reactive protein of 241 (< 5 mg/L), D-dimers of 1023 ng/mL (< 500), Na+ of 131 mmol/L, and creatinine of 1.33 mg/dL with eGFR of 59 mL/min, and hepatic enzymes were within the normal range. Chest X-ray showed a left-sided pulmonary consolidation. Chest CT scan showed a left upper lobe consolidation (Figure 1). Point-of-care PCR for flu viruses and SARS-CoV-2 was negative. Rapid test for urine *Legionella* antigen was negative. The patient was put on cefuroxime 1.5 gr TID. Sputum examination showed no germ on direct examination and grew no respiratory pathogen. Blood cultures remained negative. On day 3, pyrexia persisted at 40.3°C. Blood tests showed WBC of 7290 cells/μL, neutrophils of 91%, CRP of 427, PCT of 2.53 (< 0.21 μg/L), Na+ of 129 mmol/L, eGFR of 72 mL/min, and LDH of 304 UI/L (< 225); other enzymes were within the normal range. Treatment was switched to ceftriaxone 2 gr IV q24h and clarithromycin 500 mg PO BID. A multiplex PCR (TaqMan Array Respiratory Card) targeting *adenovirus, CMV, coronavirus (229E, NL63, OC43, and KU1/OC43), SARS-CoV-2, HSV1 and 2, HHV6, VZV, RSV, Enterovirus, influenza A, influenza B, human metapneumovirus, parainfluenza virus, rhinovirus, Parechovirus, Bordetella pertussis, Chlamydia psittaci, Chlamydophila pneumoniae, Coxiella burnetii, Legionella pneumophila, Mycoplasma pneumoniae, Pneumocystis jirovecii, Aspergillus fumigatus, Aspergillus flavus, Aspergillus niger,* and *Aspergillus terreus* was performed on a bronchoalveolar lavage (BAL). Result was positive for *Chlamydophila psittaci* DNA. Treatment was switched to moxifloxacin 400 mg q24h to complete a 14-day treatment. Within 48 h, fever abated, CRP dropped to 158 mg/L, and the patient felt much better. Two months later the control chest CT scan returned to normal (Figure 2).

## 3. Discussion

*Chlamydia psittaci* causes endemic avian chlamydiosis, epizootic outbreaks in mammals and isolated cases or outbreaks of human respiratory psittacosis [4, 5]. It is also called parrot fever (in relation to psittacine birds) or ornithosis, as it is a usual inhabitant of birds. Anti *C. psittaci* antibodies are found in about 11% of pet parrots [6]. However, several other animals including cattle, pigs, horses, and sheep can also host it. *C. abortus*, *C. felis*, and *C. caviae*, formerly included in the *C. psittaci* group, are endemic strains in mammals [7]. In humans, it is acquired by exposure to birds or related fomites [8]. It can also occur following exposure to mammals [7]. It mostly affects pet owners and veterinaries. Human-to-human transmission, including nosocomial transmission, has been reported [9, 10]. It is categorized by the US CDC as a category B bacteriological agent with potential for bacteriologic war. The life cycle of *C. psittaci* produces forms (elementary body) which can survive in the environment for months [11]. The incubation period lasts between 5 and 14 days. In humans, the severity of pneumonia ranges from paucisymptomatic to fatal, mostly in elderly and pregnant women [11]. Severe outbreaks in UK following importation of parrots with a fatality rate as high as 50% have been reported [5] in the 20^th^ century. *C. psittaci* is found as the causal agent in about 1% of CAP [12]. There is no specific radiological pattern, and it can present as uni or multilobar consolidation(s), or patchy infiltrates with/out pleural effusions [13]. The severity of the disease ranges from a flu-like illness (fever, myalgia, conjunctivitis, and cough) to severe, sometimes fatal, cases with hepatitis, myocarditis or endocarditis, encephalitis, and arthritis [11, 14]. A clear exposure to birds' fomites is present only in about 30% to 50% of the patients [15]. As atypical germs require a specific treatment beyond the first-line beta-lactams, some biological abnormalities might orient the clinician toward an “an atypical” germ. It remains difficult to differentiate “atypical” from typical pneumonia using clinical, biological, or imagery data [15]. This is why the empiric therapy should be guided by the severity of the case indicating monotherapy with beta-lactam or a beta-lactam + inhibitor of beta-lactamase or empiric adjunct of a respiratory fluoroquinolone or a macrolide [2]. During early follow-up, it is wise to switch to a respiratory fluoroquinolone or a macrolide if the patient does not fare better after 72 h of empiric beta-lactam ± inhibitor of beta-lactamase therapy. After lung inhalation, *Chlamydia psittaci* reaches the reticuloendothelial system via the blood and spreads to the liver, spleen, and the central nervous system. In a study of 120 cases of psittacosis, over 80% of the patients had abnormal liver tests and about 50% had severe increase in the liver cytolytic and cholestatic enzymes [16]. However, the exact mechanism of liver toxicity remains unclear. Along with direct invasion of hepatocytes, severe inflammatory syndrome and/or hypoxemic pneumonia might lead to liver damage. Myocardial injury, renal dysfunction, coagulopathy, hypoproteinemia, and venous thromboembolism are reported in severe cases [17]. Our patient did not report specific exposure to birds or their fomites. Acquisition during his trip in China, the country reporting most of the cases of psittacosis, is unlikely in respect to the incubation period. Our patient, as a chief cook, was exposed to slaughtered animals which could be the source as 11% of industry chicken in Belgium harbor *C. psittaci* [18]. A nationwide survey in Belgium showed that 55% of the cases are men mostly over 45 years old. There were 44 cases in 2017 in Belgium, double the 2015 number [19]. It is a mandatorily notifiable disease as advised by some authors [20]. The wider use of multiplex PCR in the diagnosis of atypical pneumonia and mandatory reporting might have contributed to an apparent increase in incidence. Diagnosis of human psittacosis can be made by several techniques [21]. Culture is cumbersome and seldom performed. It requires a biosafety level 3 laboratory. *C. psittaci* grows on cell cultures, in embryonating chicken eggs, or in mices [22]. Serology, mostly based on ELISA, is available but requires initial and convalescent titers to confirm the diagnosis. It is also hampered by false-positive ELISA and cross-reaction with other *Chlamydia* spp. For disease outbreak investigation, serology is deemed to be enough [23]. Nucleic acid amplification tests (NAATs) like real-time PCR or metagenomic next-generation sequencing (mNGS), performed on a BAL, is however the preferred diagnostic method for acute human psittacosis [24]. Agents of atypical pneumonia including *C. psittaci* can be diagnosed using mNGS [25]. mNGS can be performed on bronchoalveolar fluid or plasma samples [26]. However, mNGS also has limitations. It can have a long turnaround time for results. It is expensive. Moreover, while it is very sensitive, it lacks specificity. Multiple likely pathogens might be identified at the same time, mostly in bronchoalveolar fluids. Diagnosis of psittacosis is frequently overlooked so that the true incidence is likely to be underestimated [27]. The treatment consists of new tetracyclines (doxycycline, tigecycline, and omadacycline), a quinolone, or a macrolide usually administered for 14 days. There are reports of macrolide treatment failure, and tetracyclines are considered the preferred regimen [28]. Omadacycline has shown efficacy in the treatment of severe psittacosis [29]. An adequate treatment will reduce the mortality rate from 10%–20% to less than 1%. To prevent acquisition of *C. psittaci*, people in regular contacts with birds should wear protective equipment [30]. Diagnosis and treatment of *C. psittaci* in birds having frequent contacts with humans like pet parrots, singing birds in competitions, and birds in exhibitions should be enforced [31].

## 4. Conclusion

We report the case of a man diagnosed with human psittacosis. He did not report contact with live birds but worked as a chief cook, potentially exposed to chicken fomites. Initially severe, his pneumonia responded well to a respiratory quinolone with complete recovery. *C. psittaci* is the reported cause of about 1% of CAP. It is probably underestimated as the carriage in birds and poultry is high. Diagnosis relied mainly on NAAT or mNGS. Mandatory reporting would permit a better representation of the burden of this disease. Wearing personal protective equipment while manipulating birds and/or diagnosis and treatment of psittacosis in pet birds should be enforced to limit the spread of this, sometimes life-threatening, zoonosis.

## Data Availability

The data that support the findings of this study are available from the corresponding author upon reasonable request.
